# Religiosity and Depression at Midlife: A Prospective Study

**DOI:** 10.3390/rel12010028

**Published:** 2020-12-31

**Authors:** Micheline R. Anderson, Priya Wickramaratne, Connie Svob, Lisa Miller

**Affiliations:** 1 Spirituality Mind Body Institute, Teachers College, Columbia University, New York, NY 10027, USA;; 2 Department of Psychiatry, College of Physicians and Surgeons, Columbia University, New York, NY 10032, USA;; 3 Division of Epidemiology, New York State Psychiatric Institute, New York, NY 10032, USA; 4 Mailman School of Public Health, Columbia University, New York, NY 10032, USA

**Keywords:** depression, religiosity, spirituality, midlife, Major Depressive Disorder

## Abstract

**Objectives:**

Previously, authors found high personal importance of religion/spirituality (R/S) in early adulthood to predict a 75% decreased risk of recurrence of major depression in middle adulthood. Here, the authors follow up the original study sample to examine the association between R/S and major depression from middle adulthood into midlife.

**Method:**

Participants were 79 of 114 original adult offspring of depressed and non-depressed parents. Using logistic regression analysis, three measures of R/S from middle adulthood (personal importance, frequency of religious service attendance, and denomination) were used to predict Major Depressive Disorder (MDD) in midlife.

**Results:**

High R/S importance in middle adulthood was prospectively associated with risk for an initial onset of depression during the period of midlife. Frequency of attendance in middle adulthood was associated with recurrence of depression at midlife in the high-risk group for depression, as compared to the low-risk group.

**Conclusion:**

Findings suggest that the relation between R/S and depression may vary across adult development, with risk for depression associated with R/S at midlife potentially revealing a developmental process.

## 1. Introduction

In the past two decades, inquiries into the association between religiosity and spirituality (R/S) and mental health have provided evidence for substantial salutary effects of R/S on physical and mental health (Koenig et al. 2012). Although conflicting reports do exist (Maselko and Buka 2008; Pargament et al. 2004; Lawrence et al. 2016; Braam et al. 2007), this positive association is often confounded within retrospective or cross-sectional data as a result of individuals with psychiatric symptoms seeking out religion as a means of coping. Despite these findings, a substantial body of literature supports an inverse association between R/S and depression that remains consistent across a myriad of cross-sectional and longitudinal studies (for review, see Sharma et al. 2017; Bonelli et al. 2012; Koenig 2012a).

Reviews and meta-analyses of the literature examining this relation suggest that the majority of prospective cohort studies associated R/S with lower levels of depression or faster remission from depression, with an average effect size of the protective benefit of R/S against depression of −0.18 (Braam and Koenig 2019; Smith et al. 2003). Although relatively small, Koenig (2012a) compares this benefit to the magnitude of risk conferred by gender on depressive symptoms. Attempts to disentangle the salutogenic and depressogenic mechanisms of R/S have been largely aimed at cognitive and behavioral mechanism such as beliefs or coping strategies (positive or negative) that either aid in or deter from navigating ecological challenges. In this regard, this study and its most recent predecessor (Miller et al. 2012) establish temporality as a means to clarify the relationship between R/S and onset or recurrence of Major Depressive Disorder (MDD) in an intergenerational sample during distinct developmental periods, such as midlife.

Across this substantial body of literature, there exists little research on the stability of or change in the relationship between R/S and depression across the lifespan. The current report is part of a larger 30 year longitudinal study (Weissman et al. 2016a, 2016b) to investigate the trajectory of the relationship between R/S and MDD across the course of development, focusing here on midlife. Previously, the association between R/S and depression was assessed cross sectionally at early adulthood *(mean age at assessment of MDD* = *26*, Miller et al. 1997) and prospectively from early adulthood through the child-rearing years of middle adulthood *(mean age at assessment of MDD* = *37*, Miller et al. 2012) of this ongoing 30 year study of families at high and low risk for depression (Weissman et al. 2016a). In both reports on R/S, importance of R/S offered protective benefits against MDD, putting those with self-reported high importance of R/S at one-tenth and one-fourth the risk for MDD in early adulthood and child-rearing years of middle adulthood, respectively. From a lifespan perspective, it has been argued that the association between R/S and depression is of particular relevance during vulnerable developmental periods (i.e., adolescence and older adulthood) (Miller 2013; Peteet et al. 2018; Phillips et al. 2009), thus making re-examination of these associations at midlife a compelling addition to the literature.

Recent epidemiological assessments suggest that midlife confers the highest rate of lifetime prevalence of MDD, with women and those who were widowed/separated/divorced at increased risk (Hasin et al. 2005). Lifetime prevalence rates of MDD during this stage of life range from 16% to 22% (Hasin et al. 2005; Kessler et al. 2010). Risk factors for depression in midlife include poor quality of relationships, smoking, low self-esteem, functional impairment due to disability or illness, and lifetime and/or family history of depression (Bakhshaie et al. 2015; Barkow et al. 2003; Crowell et al. 2014; Orth et al. 2016; Teo et al. 2013; Zeiss et al. 1996). The shift into midlife often brings with it changes in the family dynamic including children leaving the home, children returning home, dissolution of marriages and conflicting demands on time including work and caregiving, which are all associated with depressive symptoms or diagnosis (Marks 1998; Dennerstein et al. 2004; Kessler et al. 2004; Sbarra et al. 2014).

Risk for recurrence of depression at midlife appears to peak in midlife for women, but in older age for men (Draper and Low 2009). Differential risk by gender for new onset and recurrence may be conferred by the perimenopause for women (Bromberger et al. 2015; Cohen et al. 2006), although mixed findings suggest that this risk is not solely conferred by biological changes (Gibbs et al. 2012; Hickey et al. 2012; Soares 2013). Family and lifetime history of depression may confer risk above and beyond other biological risks during this period (Colvin et al. 2014).

Little is known about whether the reported protective benefits of R/S remain consistent across the lifespan, or whether developmental effects might be seen through middle adulthood in a dynamic relationship with depression. For lack of longitudinal research to date, a relationship between R/S and depression has yet to show nuance or capture change across time. In a cross-sectional study that gathered data on past and current religious activity, shifts in religious activity from childhood to adulthood was associated with lifetime psychiatric diagnoses (Maselko and Buka 2008). Limitations of this study included lack of information on familial risk for depression. One cross-sectional study evidenced differential associations by gender between spirituality and depression at midlife, with men’s (but not women’s) self-report of level of spirituality inversely associated with depression (Ellermann and Reed 2001). Two longitudinal studies examining R/S and depression at midlife have conflicting findings. The first showed that R/S is protective against depression and is associated with mental well-being at midlife (Dillon and Wink 2007), while the other found that R/S confers risk for depression at midlife, but only when spirituality is reported as greater than religiosity (Vittengl 2018). Although there is no consensus among the few studies that examine R/S and depression at midlife, there are numerous studies showing the protective benefits of R/S against depression in older adults (Braam et al. 2001; Cruz et al. 2009; Koenig 2007; Li et al. 2016; Sun et al. 2012; Hayward et al. 2012). As mental health at midlife is a great predictor of physical and mental health in older adulthood, it is important that this relationship is explored as thoroughly as it has been in geriatric samples.

In summary, there are few longitudinal studies examining the association between R/S and depression at midlife and none that have examined this relationship prospectively across familial risk groups and at multiple time points across the lifespan. With the roles and developmental changes unique to this stage and contributing to increased risk for depression an understanding of whether R/S is protective against MDD at midlife is prudent. To address this gap in the literature, we pose the following research questions:

1. Is R/S associated with onset or recurrence of depression at midlife?
2. Does the association between R/S and depression at midlife differ by familial risk for depression?

## 2. Methods

The data for this study come from assessments at year 20 and year 25/30 of a 30 year longitudinal study of individuals at high and low risk for major depression. The participants, offspring at high risk and low risk for depression (based on parental status), were part of a previous study (Miller et al. 2012) and were interviewed at their most recent assessment time point. The majority of subjects were last assessed at year 30 (n = 63, M = 49 years). A smaller subset of the sample (n = 16, M = 40 years) was offered the opportunity to participate in an imaging study at year 25. As not all of the original sample returned for the year 30 assessment, these subsets of the original sample were combined and the time point is referenced henceforth as year 25/30. This study was approved by the Institutional Review Board at both Yale University and Columbia University/New York State Psychiatric Institute.

The original study consisted of probands with moderate to severe MDD who received outpatient psychopharmacologic treatment of depression. Healthy matched subjects were simultaneously selected from a sample of adults with no psychiatric history from the same community. Full methodological details have been published elsewhere (Weissman et al. 1997, 2006; Weissman et al. 2016a) and are summarized here. The original probands were mainly working-class or middle-class Caucasians from the New England area to reduce heterogeneity, as was customary when this study commenced. At each wave, assessments were conducted by clinical interviewers who were blind to the proband’s clinical status and the subject’s clinical history. Biological offspring were then followed. Offspring with one or more parent with MDD were identified as high risk (HR) and offspring with no parents with MDD were identified as low risk (LR).

### 2.1. Study Participants

Study participants were a subset of the original offspring subjects from the studies at year 10 and year 20. At year 10, 151 offspring were eligible for this study. At year 20, a final group of 114 offspring were eligible for this study if they participated in this study at both year 10 and year 20, reported on their R/S consistently at both time points and answered three items on R/S (refer to Assessments below) at both time points, and were assessed for psychiatric diagnoses at year 20. At the time, those offspring who did not report their denomination as Catholic or Protestant were excluded to keep consistent with the initial study in which denomination group was used a grouping variable (Miller et al. 1997). Of the 114 subjects who were included in the previous sample 35 did not participate in the year 25/30 assessment (79/114 = 69.2%): 25 declined, 7 could not be located or scheduled, and 3 did not complete questions on R/S at year 25 or year 30. The 79 participants included in the sample and the 35 who did not participate were compared on gender, age, risk status, MDD status between year 10 and year 20, lifetime history of depression at year 20, and religiosity variables at year 20. The only significant difference between groups was that of frequency of service attendance (35 = 40%, 79 = 63%, χ^2^ = 5.34, *p* = 0.02), such that individuals who dropped out were less likely to attend services at least once a month than those who stayed in.

### 2.2. Assessments

Major depression was assessed at all time points with the Schedule for Affective Disorders and Schizophrenia Lifetime Version (SADS) for adults (Mannuzza et al. 1986). A diagnosis of MDD at year 20 was defined as a major depressive episode between year 10 and year 20 and a diagnosis of MDD at year 25/30 was defined as a major depressive episode between year 20 and year 25 or year 30, respectively.

Assessment of R/S at year 20 and year 25/30 was based on three self-report items: (1) personal importance of religion/spirituality (“How important to you is religion or spirituality?” with response options ranging from 1 [not important at all] to 4 [highly important]); (2) frequency of attendance (“How often, if at all, do you attend church, synagogue, or other religious or spiritual services?” with five response choices ranging from “never” to “once a week or more”); and (3) current religious denomination (“How would you describe your current religious beliefs? Is there a particular denomination or religious organization that you are part of?” with 10 denominations specified, including an opportunity to specify others. R/S was composed of these three variables in the analyses at year 20 and year 25/30. Personal importance of R/S and frequency of attendance were dichotomized into binary variables to remain consistent with previous studies (Miller et al. 1997, 2012). Personal importance was split into high importance (ratings equal to 4) and low importance (ratings <4). Frequency of attendance was split into high frequency (≥1 time per month) and low frequency (<1 time per month).

### 2.3. Interviewers and Best-Estimate Procedure

Masters- and doctoral-level mental health workers who were blind to parents’ clinical status and any previous history, including religion variables, administered diagnostic clinical interviews. Multiple informants and sources were used, including first-person and corroborative interviews and medical records. All final diagnoses were based on a best-estimate procedure (Leckman et al. 1982) by either a psychiatrist or a Ph.D. psychologist who was blind to the risk status and was not involved with interviewing.

## 3. Statistical Analysis

McNemar tests were used for preliminary analyses of within-group differences in demographic variables, rates of depression, and R/S variables in the sample group between year 20 and year 25/30. Chi-square tests were used for between-group differences between risk groups at year 25/30. Logistic regression analyses examined religiosity as a prospective protective factor against major depression. Year 25/30 major depression was the dichotomous outcome variable and each of the year 20 religiosity variables were used individually to conduct univariate regression. Age, sex, parental depression (risk) status, and lifetime history of MDD were included to control for potential confounds.

As previous findings have shown differential associations by risk and by lifetime history of depression, we sought to determine whether risk for depression (based on parental lifetime depression status) moderated the effect of R/S on subsequent recurrence and new onset of depression. The data were stratified both by risk status and lifetime history of depression (yes/no). Within these stratified groups, logistic regression analyses examined the three R/S variables at year 20 as predictors of the outcome of depression at year 25/30. Age and gender were included as covariates in the analysis. All statistical analyses were performed using SPSS Statistics version 20 for MAC.

## 4. Results

### 4.1. Sample Characteristics

Table 1 summarizes the demographic and clinical variables and responses to the three religiosity items at year 25/30 for the 79 participants whose data were included in the analysis. The data were stratified by risk. In the period between year 20 and year 25/30 there was an overall increase in the lifetime rate of depression (year 20 = 51%, year 25/30 = 58%, *p* = 0.03), a decrease in the rate of frequent attendance (year 20 = 63%, year 25/30 = 43%, *p* < 0.001), and changes in marital status (year 20, married = 62%, year 25/30, married = 75%. *p* = 0.03). The rates of marital status, income, and education did not differ between risk groups at year 25/30.

The rate of lifetime major depression at year 20 was twice as great in the high-risk group as the low-risk group (61% [33/54] compared with 28% [7/25]; χ^2^ = 7.50, df = 1, *p* < 0.01). However, prevalence of major depression between year 20 and year 25/30 did not differ significantly between the high-risk group and the low-risk group (37% [20/54] compared with 24% [6/25]). Although the rates of importance and attendance were consistently greater at year 20 and year 25/30 in the low-risk group as compared to the high-risk group, these differences were not statistically significant.

### 4.2. Religiosity and Major Depression

Table 2 lists, for each of the three R/S variables, the odds ratios for major depression at 25/30 year follow up while controlling for age, sex, risk status, and history of depression of offspring in the full sample. Participants with high personal importance of R/S compared with other participants had approximately 4 fold the risk of having an episode of major depression between year 20 and year 25/30 (OR = 4.10, 95% CI = 1.14–14.73, *p* = 0.03). Frequent attendance at religious services at year 20 was significantly associated with the odds of major depression between year 20 and year 25/30, such that participants who attended religious services at least once a month were 4 fold as likely to experience a major depressive episode than those who did not (OR = 4.07, 95% CI = 1.05–15.70, *p* = 0.04). Religious denomination at year 20 was not associated with MDD at year 25/30.

### 4.3. Recurrence versus New Onset of Major Depression

Table 3 lists the odds ratios for recurrence and new onset of major depression at 25/30 year follow up while controlling for age and sex of offspring for each of the three religiosity variables. Participants with no previous history of depression and reported high personal importance of R/S had approximately 14 fold the risk of having an episode of major depression between year 20 and year 25/30 (OR = 14.42; 95% CI = 1.06–196.51, *p* = 0.05), regardless of risk status. For participants with a previous history of depression, there was a significant association between frequent attendance at year 20 and recurrence of major depression between year 20 and year 25/30 (OR = 6.44; 95% CI =1.13–36.65, *p* = 0.04). When stratified by risk (data not shown), this association held only in the HR group (OR = 9.54; 95% CI = 1.18–77.32, *p* = 0.04). To test the possibility that individuals with recurrent depression at year 25/30 were more likely to be frequent attenders at year 20 because they were more likely to be depressed between year 10 and year 20, we examined the association between MDD at year 20 (defined as an episode of MDD between year 10 and year 20) and attendance at year 20. If this were true, we would expect to find that participants with MDD between year 10 and year 20 would be more likely to attend religious services at year 20; however, we found no significant association between these two variables at year 20.

## 5. Discussion

With the shift into midlife in our longitudinal study sample, we found an overall increase in the lifetime rate of depression, a decrease in the frequency of attendance at religious services (≥1 time per month), and significant changes in marital status in the entire sample between year 20 and year 25/30. When divided by familial risk for depression (HR and LR), rates of diagnosis of MDD between year 20 and year 25/30 (new onset and recurrence combined) did not differ significantly as compared to significantly different lifetime rates of MDD between HR and LR groups at year 20, with the HR group having significantly higher rates of depression than the LR group. Rates of importance of R/S and frequent attendance did not differ significantly between the groups. Importance of R/S and frequent attendance of religious services at year 20 predicted MDD at year 25/30. When the group was divided based on both lifetime history of MDD (yes/no) and risk status (HR/LR), importance of R/S at year 20 was predictive of new onset of MDD at year 25/30 across groups. Attending religious services at least once a month was predictive of recurrence of MDD between year 20 and year 25/30 uniquely in the HR group.

### 5.1. Research Questions

#### 5.1.1. Is R/S Associated with Depression at Midlife?

In this sample, regardless of risk status, the importance of R/S at year 20 was associated with a 14-fold increased risk of new onset of MDD at year 25/30. Frequency of attendance and denomination did not predict a new onset, suggesting that importance of R/S confers unique risk (among these three measures of R/S) for depression at midlife for those who have never experienced a depressive episode before. In that the importance of R/S previously was found in the same sample to be protective against depression during early adulthood and middle adulthood and there is a growing body of evidence to suggest a similar association in older adulthood as well (Koenig 2017), these longitudinal findings may suggest a *changing* relationship between R/S and depression across adult development, with a passage of risk for depression bracketed at midlife.

That developmental risk for new onset of depression was 14 fold for those who reported that R/S was important is in contrast with a small body of research on R/S at midlife, which suggests that R/S is associated with mental well-being at midlife (Ellermann and Reed 2001; Dillon and Wink 2007). Importantly, the self-rated level of R/S in this sample is, on average, much higher than in Dillon and Wink’s sample. Taken with the previous findings that dimensions of R/S have been protective in this sample, individuals’ wellbeing in this sample may be closely tied to a personal importance of R/S. We can conjecture that, alongside this stable personal importance of R/S, protective benefits may be mitigated and even for a passage in time reversed via aspects of R/S identified (but not measured here) as mediators of a direct association between R/S and depression such as intrinsic religiosity, negative religious coping, or spiritual struggle (Abu-Raiya et al. 2015; Braam et al. 2014; Pargament et al. 2004; Pearce and Koenig 2016; Smith et al. 2003; Sternthal et al. 2010; Pirutinsky and Rosmarin 2018) if potentially stressed by specific developmental challenges, losses or responsibilities endemic to midlife. These studies and others suggest that highly religious/spiritual individuals, stressed under the conditions of negative life events, may be more likely to face spiritual struggle and depression.

From a developmental perspective, there has been a general consensus among classical and modern theorists that midlife is a time when individuals develop an increased interiority, reflectiveness, awareness of themselves in space and time, a renewed desire for balance in work and leisure, and a potential for integration of the self that exceeds that of prior developmental stages (Levinson 1978, 2011; Havighurst 1948; Neugarten 1968). Some theorists have included spiritual growth as part of a process of the integration of a differentiated self, emerging via confronting and overcoming a complex environment (Bianchi 2011; Jung 1952; Neugarten 1968). Those theorists focused specifically on R/S development have suggested that midlife is a time during which the individual faces existential voids and conflict, yet can be propelled through this process to reach a deeper transcendence, appreciation of life, and relationship with G-d or their Higher Power (Fowler 1981; Jung 1960). The R/S challenges inherent in midlife ultimately can be transformative, perhaps even requisite for maturation of faith (Koenig 1994). Qualitative data suggest that R/S development includes passages that are arduous, non-linear, and extends beyond the self to include and interact with interpersonal and relational facets of life (Ray and McFadden 2001; Russo-Netzer and Mayseless 2017). This non-linear development of R/S mirrors research on the trajectory of R/S over the lifespan that suggests: R/S often emerges through struggle across adolescence and emerging adulthood, is highly protective throughout adulthood, and then undergoes a second wave of emergence through struggle from midlife that is highly protective in late adulthood (Desrosiers and Miller 2007; Dillon and Wink 2007; Wink and Dillon 2002). The data from the present study may reflect the impact of incumbent challenges in midlife and process of struggle and emergent growth as it relates to R/S at this stage.

It is noteworthy that individuals with the strongest spirituality and religiousness struggle the most at midlife. This developmental phase of R/S might reflect the notion shared across religious traditions of a “dark night of the soul,” a time when the more spiritually connected experience challenges or felt distance more acutely, leading in time to a deepening of faith. A follow-up study on this sample on the other side of midlife would **further** address this potential process.

#### 5.1.2. Does the Association between R/S and Depression at Midlife Differ by Risk Group?

Although the association between importance of R/S did not confer a differential risk for depression between HR and LR groups, we found that frequency of attendance at year 20 was associated with an increased risk of recurrence of depression in midlife by 10 fold in the HR group. Longitudinal data on the original depressed probands of this study illustrate that the protective effect of church attendance has rather been associated with risk when controlling for other R/S variables at midlife (Miller et al. 1997), suggesting a familial susceptibility to recurrent depression that offsets any protective capacity of frequent attendance at this stage. Alternately, individuals at HR for depression may seek out companionship or social support through religious community (Ano and Vasconcelles 2005; Ellison et al. 1994; Holt et al. 2013), which may explain this association.

### 5.2. Clinical Implications

The protective benefits of R/S need to be seen in a long-term developmental trajectory with passages of R/S struggle, emergence and strengthening. The majority of studies to date are cross sectional. Most studies show a strong protective benefit of R/S against depression, while some studies have shown R/S to be a risk factor for depression either 1) during phases of emergent development such as adolescence or midlife, or 2) under potentially distorting formative conditions of parental psychopathology or developmental psychopathology (Gur et al. 2005; Miller et al. 2002; Sorenson et al. 1995) or when missing central protective qualities such as connection to God (Kendler et al. 1997; Pargament et al. 2005).

A host of treatment models have been published that address R/S struggles, emergence and strengthening within psychotherapy (Koenig 2012b; Koszycki et al. 2014; Pargament 2007; Pearce et al. 2015; Santiago and Gall 2016; for reviews see Hodge 2006; McCullough 1999; Smith et al. 2007). Awareness of the developmental R/S across adulthood in mental health treatment is essential, as some studies suggest that some religious individuals avoid psychotherapy due to both perceptions about and actual tendencies of clinicians to in marginalize or exclude faith beliefs in the treatment process (Bergin 1991; Delaney et al. 2007; Hathaway et al. 2004; Mayers et al. 2007; Richards and Bergin 2000; Shafranske and Gorsuch 1984). This, in combination with recent findings that more than half of treating clinicians do not feel competent addressing patients’ religiosity and spirituality (Vieten et al. 2013, 2016) suggests that efforts to bridge the gap between patient beliefs and clinician readiness should focus on aspects of clinical training.

### 5.3. Limitations

To the best of our knowledge, the current study is the first longitudinal study to assess the changing relationship between R/S and depression at midlife, potentially shedding light on spiritual struggle at midlife, particularly among people with a strong personal R/S. Study limitations include difficulty in generalizing these findings due to the sample’s size and demographically homogeneous makeup. To remain consistent with other studies using this sample, religiosity and spirituality are not distinguished as separate variables in this study and are dichotomized. The individuals from this sample endorsed high levels of R/S and thus, the differences between the ‘high’ and ‘low’ R/S groups may be more subtle than in other studies which examine data such as these in this fashion. Further, this sample is predominantly Catholic and therefore not representative of the overall U.S. population.

In this high-risk longitudinal sample, there was a high rate of individuals with previous depressive episodes and a small number of cases of new onset of depression. We also are unable to account for mechanisms for drop out and thus selected to use listwise deletion of cases rather than impute missing data, recognizing this changes the data available to estimate model parameters. Additionally, the width of confidence intervals in the regression analyses were large and we did not adjust *p* values for multiple comparisons. Finally, the significant difference in frequency of attendance between the retained sample and those who did not participate from the previous study suggests that individuals who frequently attended religious/spiritual services were more likely to participate in this study. Thus, these findings must be interpreted with caution.

## 6. Conclusions

The current longitudinal, prospective study shows that R/S is associated with a 14-fold increase in risk for depression at midlife in the same sample of adults to have derived a 75% protective benefit against depression during early and middle adulthood. This life-course study raises the possibility of a period of spiritual struggle, a “dark night of the soul” in the changing relationship between R/S and depression during the passage of midlife.

This interpretation of an adult development model, that includes the possibility of struggle and potentially emergent growth at midlife, is raised by the longitudinal nature of this study, specifically in that the association of R/S and depression changes across the life course from offering sustained protective benefits through child-rearing years of middle adulthood, to then conferring risk across risk groups at midlife. Alongside evidence that R/S guards against depression among younger and older adults, this study may highlight a developmental effect specific to midlife that is subject to further change in future life stages. The limitations of these data highlight the need for further inquiry into the prospective risk for depression at midlife associated with R/S.

## Figures and Tables

**Table 1.** Demographic, religiosity, and clinical characteristics of 79 adult offspring of depressed and nondepressed parents at 25/30-year follow-up in a 30-year study.

Characteristic				
	Low Risk (N = 25)		High Risk (N = 54)	
	Mean	SD	Mean	SD
Age	45.8	5.4	48.2	7.4
	N	%	N	%
Female	14	56.0	35	64.8
Annual income ($)				
<20,000 (low)	1	4.0	2	3.7
20,000–39,000 (medium)	3	12.0	5	9.3
>40,000 (high)	21	84.0	43	79.6
Education level				
<High school	0	0.0	1	1.9
High school graduate	6	24.0	14	25.9
>High school	19	76.0	39	72.2
Marital status				
Single	2	8.0	5	9.3
Married	21	84.0	38	70.4
Separated or divorced	2	8.0	11	20.4
Religiosity				
Personal importance of religion/spirituality				
High	12	48.0	23	42.6
Moderate	12	48.0	20	37.0
Slight	1	4.0	7	13.0
None	0	0.0	4	7.4
Frequent attendance at religious/spiritual services^a^	13	52.0	21	38.9
Religious denomination				
Protestant	3	12.0	8	14.8
Catholic	19	76.0	34	63.0
Clinical characteristics				
Major depression^b^				
Lifetime episode (at year 20) **	7	28.0	33	61.1
Episode between year 20 and year 25/30	6	24.0	20	37.0

Notes:

a Attends at least once a month;

b Assessed with the Schedule for Affective Disorders and Schizophrenia-Lifetime Version;

** Significant difference between group at *p* < 0.01.

**Table 2.** Odds ratios of recurrence or onset of Major Depressive Disorder in 79 adult offspring of depressed and nondepressed parents between 20- and 25/30 year follow-ups associated with religiosity variables at year 20.

Religiosity Variable	Univariate Models^a^			
	Odds Ratio	95% CI	Wald χ^2^	*p*
Religion/spirituality highly important	4.10	1.14–14.73	4.69	0.03
Frequent attendance at religious/spiritual services	4.07	1.05–15.70	4.15	0.04
Catholic (vs. Protestant)	1.45	0.26–8.15	0.18	0.67

Notes:

a For each of the three univariate models, the outcome measure is Major Depressive Disorder at year 25/30 (yes/no) and the primary predictor is the dichotomous religiosity variable at year 20. Sex, age, history of depression, and risk status (high or low, based on parental depression status) are controlled.

**Table 3.** Odds ratios of recurrence and new onset of Major Depressive Disorder in 79 adult offspring of depressed and non-depressed parents between 20- and 25/30 year follow ups associated with religiosity variables at year 20.

	Recurrence^a,b^				New Onset^c,d^			
Religiosity Variable	Odds Ratio	95% CI	Wald χ^2^	*P*	Odds Ratio	95% CI	Wald χ^2^	*p*
Religion/spirituality highly important	2.44	0.57–10.46	1.44	0.23	14.42	1.06–196.51	4.01	0.05
Frequent attendance at religious/spiritual services	6.44	1.13–36.65	0.04	0.04	1.41	0.19–10.52	0.11	0.74
Catholic (vs. Protestant)	0.57	0.06–4.95	0.27	0.61	5.6*10^8^	0.00	0.00	0.99

Notes:

a For this model, the outcome measure is recurrence of MDD, as defined by having a previous episode of MDD prior to the period between year 20 and year 25/30. Sex and age are controlled.

b There were 20 individuals with a recurrence of a depressive episode between years 20 and 25/30.

c For this model, the outcome measure is new onset of MDD, as defined by having no episodes of MDD prior to the period between year 20 and year 25/30. Sex and age are controlled.

d There were 6 individuals with new onset of a depressive episode between years 20 and 25/30.

## Data Availability

Data were obtained as part of an ongoing, multi-generational study of families at risk for depression that started in 1982 (before data sharing existed); therefore, consent was not obtained. According to the Institutional Review Board at Columbia University and New York State Psychiatric Institute, public data sharing, even anonymously, is restricted by participants’ informed consent.
